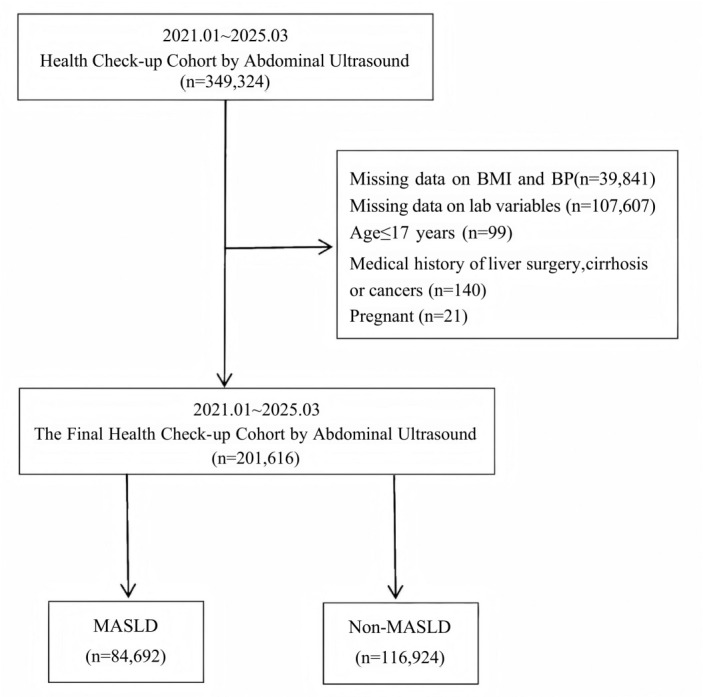# Epidemiology of metabolic dysfunction-associated steatotic liver disease and discordance in non-invasive fibrosis scores in Eastern China: A cross-sectional study

**DOI:** 10.1097/MD.0000000000049110

**Published:** 2026-06-05

**Authors:** Mingxing Chang, Peipu Shen, Guifang Shen

**Affiliations:** a Department of Health Management Center, The Affiliated Hospital of Xuzhou Medical University, Xuzhou, Jiangsu, China.

**Keywords:** FIB-4, liver fibrosis, metabolic dysfunction-associated steatotic liver disease, noninvasive tests, prevalence

## Abstract

The contemporary epidemiology of metabolic dysfunction-associated steatotic liver disease (MASLD) and the risk stratification provided by noninvasive tests (NITs) for fibrosis in large populations require further characterization. Specifically, the degree of agreement between different NITs and how their association with metabolic comorbidities varies in Chinese MASLD cohorts remains unclear. This study aimed to systematically evaluate the prevalence of MASLD and to compare the performances among 3 NITs for fibrosis in a large eastern Chinese cohort. In this cross-sectional study, which included 201,616 adults from eastern China between January 01, 2021 and March 31, 2025, MASLD was diagnosed based on hepatic steatosis on ultrasonography plus metabolic criteria according to the latest guidelines. Liver fibrosis was assessed using the FIB-4 index, nonalcoholic fatty liver disease fibrosis score (NFS), and aspartate transaminase to platelet ratio index (APRI). Sex-and age-standardized prevalence was calculated. Associated factors were analyzed via multivariable logistic regression. The standardized prevalence of MASLD was 39.42%, higher in males (49.98%) than females (28.59%), with obesity as the strongest risk factor (aOR: 7.69). Among MASLD patients, significant fibrosis prevalence was similar when assessed by FIB-4 (19.12%) and NFS (21.32%); advanced fibrosis was lowest according to APRI (0.61%) compared to FIB-4 (1.79%) and NFS (1.97%). Critically, the associations between metabolic comorbidities (e.g., diabetes, dyslipidemia) and fibrosis risk varied substantially depending on the NIT used. MASLD is highly prevalent in this eastern Chinese health checkup cohort. The marked discordance in fibrosis risk stratification across different NITs, particularly in patients with metabolic comorbidities, highlights that these tools provide complementary rather than interchangeable information. These findings suggest the need for cautious interpretation of NIT results and further research into context-aware risk stratification strategies that consider both the specific NIT employed and the patient’s metabolic profile.

## 1. Introduction

Metabolic dysfunction-associated steatotic liver disease (MASLD) has emerged as a major and growing global public health issue. Its redefinition from nonalcoholic fatty liver disease (NAFLD) highlights the primacy of metabolic dysregulation,^[1]^ requiring updated epidemiological assessments, especially in China where metabolic risk factors are highly prevalent.^[2,3]^ While previous studies have focused on NAFLD, the epidemiology and disease burden of MASLD under its new, metabolism-centric definition remain incompletely characterized.

A central priority in managing MASLD is identifying patients at risk of advanced liver fibrosis – the key predictor of liver-related outcomes.^[4]^ Early identification of significant (SF) or advanced fibrosis (AF) is crucial, as early-stage changes may be reversible with intervention, whereas progression to cirrhosis significantly increases the risk of complications.^[5]^ As liver biopsy is invasive and unsuitable for population-level screening, and advanced imaging-based elastography is often logistically or economically unfeasible for large-scale use, current guidelines recommend noninvasive tests (NITs) such as the FIB-4 index, NAFLD fibrosis score (NFS), and aspartate aminotransferase (AST) to platelet ratio index (APRI) for initial risk stratification.^[1,6]^ This study was designed to compare the real-world application of these widely available NITs in a large Chinese MASLD cohort and to assess the level of agreement between them, aiming to inform their practical application in resource-conscious settings..

Although NITs have been used to estimate fibrosis burden in Western populations,^[7,8]^ robust evidence on their systematic use and, importantly, their direct comparability in large Chinese MASLD cohorts remains limited. This gap impedes an accurate assessment of the national fibrosis burden and hinders public health planning. Moreover, since these NITs incorporate different clinical variables, their performance may vary across patient subgroups.^[9]^ A direct, large-scale comparison in a real-world MASLD population is essential to clarify these potential disparities and guide the development of refined, context-aware screening strategies.

Furthermore, MASLD prevalence exhibits substantial sex and age heterogeneity, linked to factors such as sex hormones, body fat distribution, and metabolic changes over the life course.^[1]^ Determining the sex- and age-standardized prevalence and distribution patterns of MASLD in China is therefore critical for developing targeted public health strategies and identifying high-risk subgroups. To address these concurrent evidence gaps, we conducted a large-scale study involving 201,616 adults from eastern China. The study had 2 primary objectives: first, to determine the sex- and age-standardized prevalence of MASLD; and second, to describe the prevalence of NIT-defined fibrosis risk using validated noninvasive biomarkers, while directly comparing the risk stratification provided by FIB-4, NFS, and APRI and quantifying the agreement between them, as well as identifying key determinants associated with NIT-defined high-risk status as defined by each tool.

## 2. Materials and methods

### 2.1. Study population and design

We conducted a cross-sectional study of adults (≥18 years) who underwent health checkups with abdominal ultrasonography at our institution in eastern China. The study period spanned from January 01, 2021 to March 31, 2025. Exclusion criteria were: incomplete data for MASLD diagnosis or fibrosis assessment; established cirrhosis, hepatocellular carcinoma, or liver surgery; severe cardiorenal dysfunction; and pregnancy. For individuals with multiple visits, one record was randomly retained to prevent duplication. Ultimately, 201,616 participants were consecutively enrolled (see Fig. S1, Supplemental Digital Content 1, which illustrates the study flow diagram). Assuming an expected MASLD prevalence of 30 to 35%,^[1]^ a 95% confidence level, and a desired precision of ±1%, the minimum required sample size was approximately 8065 to 8746 participants. After adjusting for 10% incomplete data, we targeted 8872 to 9621 participants. The final sample of 201,616 far exceeds this requirement. The study adhered to the Strengthening the Reporting of Observational Studies in Epidemiology (STROBE) guidelines. The study was approved and supervised by the Ethics Committee of the Affiliated Hospital of Xuzhou Medical University (Approval Number: XYFY2023-KL086-01). The study was performed in accordance with the ethical standards of the Declaration of Helsinki. The need for written informed consent was waived by the Ethics Committee due to the retrospective analysis of de-identified data from health checkup archives. Throughout the data collection, analysis, and manuscript preparation, the authors had no access to information that could identify individual participants, as the dataset provided for research was fully anonymized.

### 2.2. Data collection

Trained medical staff collected demographic characteristics, medical history, and conducted physical examinations following standardized protocols. Anthropometric measurements included height, weight, systolic blood pressure, and diastolic blood pressure. Body mass index (BMI) was calculated as weight in kilograms divided by height in meters squared (kg/m^2^). The biochemical profile included fasting blood glucose (FBG), total cholesterol, triglycerides (TG), high-density lipoprotein cholesterol (HDL-C), low-density lipoprotein cholesterol (LDL-C), alanine aminotransferase (ALT), AST, blood urea nitrogen, creatinine, uric acid, albumin, and platelet count (PLT). All assays were performed using standardized automated analyzers. Hepatic steatosis was diagnosed via abdominal ultrasonography using standardized ultrasound scanners (Affiniti70, Philips Medical Systems, Best, the Netherlands) based on established ultrasonographic criteria.

### 2.3. Definitions of MASLD and various subpopulations

MASLD Diagnosis: Hepatic steatosis was diagnosed via abdominal ultrasonography performed by trained radiologists using standard criteria. MASLD is defined by the presence of hepatic steatosis alongside at least one cardiometabolic risk factor, after exclusion of other liver disease etiologies and significant alcohol intake, as proposed by the recently published guidelines.^[1]^ The key cardiometabolic criteria, adapted for the Chinese population, are: BMI ≥ 23 kg/m^2^ or waist circumference ≥ 90/80 cm for males/females; blood pressure ≥ 130/85 mm Hg or ongoing antihypertensive treatment; TG ≥ 150 mg/dL (1.70 mmol/L) or triglyceride-lowering treatment; and HDL-C ≤ 40/50 mg/dL (1.0/1.3 mmol/L) for males/females or lipid-lowering treatment.

Study subpopulations were categorized based on demographic and clinical characteristics as follows: age (<60 years, ≥60 years), sex (male, female). BMI was classified according to Asian-specific criteria,^[10]^ including underweight (<18.5 kg/m^2^), normal (≥18.5 to <23 kg/m^2^), overweight (≥23 to <25 kg/m^2^) and obesity (≥25 kg/m^2^). Type 2 diabetes was defined as FBG ≥ 7.0 mmol/L, self-reported physician diagnosis, or current use of glucose-lowering medication.^[11]^ Hypertension was defined as systolic blood pressure ≥ 140 mm Hg and/or diastolic blood pressure ≥ 90 mm Hg, self-reported diagnosis of hypertension, or current use of antihypertensive drugs.^[12]^ Dyslipidemia was defined according to the guidelines for the prevention and treatment of dyslipidemia in Chinese adults,^[13]^ meeting at least one of the following criteria: total cholesterol ≥ 5.2 mmol/L, LDL-C ≥ 3.4 mmol/L, HDL-C < 1.0 mmol/L, TG ≥ 1.7 mmol/L. Elevated liver enzymes were defined as ALT > 40 U/L or AST > 40 U/L.

### 2.4. Assessment of liver fibrosis

Liver fibrosis was assessed using 3 validated noninvasive indices: the FIB-4 index, NFS, and APRI. The respective formulas are as follows:

FIB-4 Index = (age [years] × AST [U/L])/ (PLT [×10^9^/L] × √ALT [U/L])
NFS = −1.675 + 0.037 × age (years) + 0.094 × BMI (kg/m^2^) + 1.13 × impaired fasting glucose/diabetes (yes = 1, no = 0) + 0.99 × AST/ALT ratio − 0.013 × PLT (×10^9^/L) − 0.66 × albumin (g/dL).
APRI = [(AST [U/L]/ AST ULN)/ PLT (×10^9^/L)] × 100, where AST ULN = 40 U/L. (ULN: Upper Limit of Normal)

Fibrosis stages were defined as follows^[14]^:

AF (AF, corresponding to histological stage ≥ F3) was defined as FIB-4 > 2.67, NFS > 0.676, APRI > 1.0
SF (SF, corresponding to histological stage ≥ F2) was defined as FIB-4 between 1.30 to 2.67 or NFS between–1.455 to 0.676
Low risk was defined as FIB-4 < 1.30, NFS < −1.455, and APRI < 1

### 2.5. Sex- and age-standardized prevalence

To obtain population-representative estimates, we calculated sex- and age-standardized prevalence based on the 2020 Chinese national census data. The standardization was performed across 7 age groups. Since the census data use 5-year intervals starting from 15 to 19 years, we adapted the grouping to maintain epidemiological rigor while ensuring statistical stability. Participants aged 18 to 19 years were combined with the 20 to 29 years group to form an “18 to 29 years” category. Subsequent groups followed conventional 10-year intervals: 30 to 39, 40 to 49, 50 to 59, 60 to 69, 70 to 79, and ≥80 years. The sex- and age-standardized prevalence of MASLD was estimated. Additionally, the prevalence of SF and AF among MASLD patients was also calculated.

### 2.6. Statistical analysis

Categorical variables are presented as numbers with percentages, and continuous variables are summarized as mean ± standard deviation or median with interquartile range, based on their distribution. Group comparisons were performed using Chi-square tests for categorical variables, Student’s t tests for normally distributed continuous variables, and Mann–Whitney *U* tests for non-normally distributed variables. Multivariable logistic regression models were constructed to identify independent associated factors for both MASLD in the overall population and fibrosis risk among MASLD patients. Separate models were developed for each NIT (FIB-4, NFS, APRI) and fibrosis stage (AF, SF). Model 1 was adjusted for sex and age only, while Model 2 was further adjusted for additional metabolic confounders, including obesity, diabetes, hypertension, dyslipidemia, elevated ALT, and elevated AST. Due to the cross-sectional design, identified associations should be interpreted as correlational and do not imply causation. Furthermore, as this study lacks histological confirmation, all references to fibrosis prevalence refer to NIT-defined risk categories, not histologically confirmed fibrosis stages. Participants with any missing data for variables in a given multivariable model were excluded from that specific analysis (complete-case analysis). All statistical analyses in this study were performed using SPSS 26.0 (IBM Corp., Armonk); a two-tailed *P* value < .05 was deemed statistically significant.

## 3. Results

### 3.1. Study population and baseline characteristics

A total of 201,616 participants (mean age 45 years; 59.7% male) were included in this cross-sectional analysis. As detailed in Table 1, participants with MASLD (n = 84,692) exhibited a distinct metabolic phenotype compared to those without MASLD. They were significantly more likely to be male, older, and presented with markedly adverse metabolic parameters, including higher BMI, blood pressure, FBG, TG, and LDL-C, alongside lower HDL-C (all *P* < .001). The MASLD group also carried a greater burden of metabolic comorbidities, including obesity, diabetes, hypertension, and dyslipidemia (all *P* < .001). Furthermore, MASLD participants demonstrated substantial hepatic involvement, characterized by an elevated liver enzyme profile and a 3- to 4-fold higher prevalence of elevated ALT and AST, respectively. This group also showed higher levels of renal function markers (blood urea nitrogen, creatinine, uric acid). The NITs (FIB-4, NFS, APRI) were significantly higher in the MASLD group, though absolute median values suggested a low probability of NIT-defined AF in the overall cohort.

**Table 1:** Characteristics of the MASLD and non-MASLD groups.

Characteristics	Overall (n = 201,616)	Non-MASLD (n = 116,924)	MASLD (n = 84,692)	*P* value
General information				
Male*	120,427 (59.7)	57,580 (49.2)	62,847 (74.2)	<.001
Age, yr†	45 (36, 57)	43 (34, 55)	48 (38, 58)	<.001
BMI, kg/m^2^‡	24.71 ± 3.57	22.94 ± 2.80	27.15 ± 3.03	<.001
SBP, mm Hg‡	127.31 ± 18.19	123.05 ± 17.54	133.18 ± 17.42	<.001
DBP, mm Hg‡	77.71 ± 11.80	74.73 ± 11.00	81.81 ± 11.62	<.001
Glucolipid metabolism				
FBG, mmol/L†	5.22 (4.89, 5.65)	5.10 (4.80, 5.45)	5.42 (5.05, 5.97)	<.001
TC, mmol/L‡	4.81 ± 1.00	4.69 ± 0.95	4.98 ± 1.02	<.001
TG, mmol/L†	1.30 (0.89, 1.94)	1.04 (0.76, 1.46)	1.79 (1.27, 2.57)	<.001
HDL-C, mmol/L†	1.31 (1.12, 1.54)	1.40 (1.21, 1.63)	1.20 (1.04, 1.38)	<.001
LDL-C, mmol/L‡	3.01 ± 0.77	2.88 ± 0.74	3.18 ± 0.78	<.001
Hepatic and renal function				
ALT, U/L†	19 (14, 29)	16 (12, 23)	25 (18, 38)	<.001
AST, U/L†	20 (17, 24)	19 (16, 22)	21 (18, 26)	<.001
BUN, mmol/L†	4.90 (4.13, 5.80)	4.79 (4.01, 5.69)	5.05 (4.30, 5.94)	<.001
Cr, μmol/L†	66 (56, 76)	63 (54, 74)	70 (60, 78)	<.001
UA, μmol/L†	319 (261, 382)	292 (243, 350)	357 (299, 418)	<.001
Albumin, g/L‡	45.76 ± 2.62	45.57 ± 2.63	46.02 ± 2.59	<.001
Platelet, ×10^9^/L†	236 (203, 274)	233 (200, 271)	240 (206, 278)	<.001
Hepatic fibrosis index				
FIB-4†	0.846 (0.601, 1.218)	0.845 (0.605, 1.228)	0.847 (0.595, 1.204)	<.001
NFS†	−2.638 (−3.432, −1.729)	−2.747 (−3.497, −1.908)	−2.468 (−3.327, −1.484)	<.001
APRI†	0.213 (0.167, 0.278)	0.202 (0.160, 0.262)	0.227 (0.178, 0.298)	<.001
Metabolic disorder				
Obesity*	90,557 (44.9)	26,315 (22.5)	64,242 (75.8)	<.001
Diabetes*	14,813 (7.3)	4765 (4.0)	10,048 (11.8)	<.001
Hypertension*	60,405 (29.9)	24,166 (20.6)	36,239 (42.7)	<.001
Dyslipidemia*	111,236 (55.1)	48,430 (41.4)	62,806 (74.1)	<.001
Elevated ALT*	24,639 (12.2)	6331 (5.4)	18,308 (21.6)	<.001
Elevated AST*	7920 (3.9)	2451 (2.1)	5469 (6.5)	<.001

Note: Percentages may not add to 100% because of rounding.

ALT = alanine aminotransferase, APRI = AST to platelet ratio, AST = aspartate transaminase, BMI = body mass index, BUN = blood urea nitrogen, Cr = creatinine, DBP = diastolic blood pressure, FBG = fasting blood glucose, HDL-C = high-density lipoprotein cholesterol, LDL-C = low-density lipoprotein cholesterol, MASLD = metabolic-associated steatotic liver disease, NFS = NAFLD Fibrosis Score, SBP = systolic blood pressure, TC = total cholesterol, TG = triglyceride, UA = uric acid.

* Data are presented as n (%).

† Data are presented as median (interquartile range).

‡ Data are presented as mean (SD).

### 3.2. Prevalence and distribution patterns of MASLD

The sex- and age-standardized prevalence of MASLD was 39.42% (95% CI: 39.16–39.68). As detailed in Table 2, the prevalence varied substantially across population subgroups, revealing a pronounced disease burden in specific demographic and metabolic categories. A strong male predominance was observed (49.98% vs 28.59%), along with a significantly higher prevalence in adults aged ≥ 60 years (48.43%) compared to younger individuals (36.61%) (all *P* < .001). Metabolic abnormalities were strongly associated with MASLD, with the highest prevalence rates exceeding 65% among individuals with obesity (67.42%) or type 2 diabetes (73.10%). The high prevalence of MASLD among individuals with elevated liver enzymes (66.12% for ALT, 61.66% for AST) underscores its hepatic impact. Furthermore, the disease burden remained substantial in those with hypertension (55.78%) or dyslipidemia (50.60%), reinforcing the close link between MASLD and broader cardiometabolic dysfunction (all *P* < .001).

**Table 2:** Prevalence of MASLD in the overall study population and subpopulations.

Populations	No. of participants (%)	Prevalence, % (95% CI)
Overall	84,692	39.42 (39.16–39.68)
Sex		
Male	120,427 (59.7)	49.98 (49.61–50.35)
Female	81,189 (40.3)	28.59 (28.22–28.97)
*P* for difference		<.001
Age, yr		
<60	163,304 (81.0)	36.61 (36.31–36.92)
≥60	38,312 (19.0)	48.43 (48.26–48.60)
*P* for difference		<.001
Obesity		
Yes	90,557 (44.9)	67.42 (66.87–67.96)
No	111,059 (55.1)	19.43 (19.18–19.68)
*P* for difference		<.001
Diabetes		
Yes	14,813 (7.3)	73.10 (71.44–74.76)
No	186,803 (92.7)	37.98 (37.73–38.23)
*P* for difference		<.001
Hypertension		
Yes	60,405 (30.0)	55.78 (55.02–56.54)
No	141,211 (70.0)	33.99 (33.68–34.31)
*P* for difference		<.001
Dyslipidemia		
Yes	111,236 (55.2)	50.60 (50.17–51.03)
No	90,380 (44.8)	26.69 (26.36–27.03)
*P* for difference		<.001
Elevated ALT		
Yes	24,639 (12.2)	66.12 (65.19–67.05)
No	176,977 (87.8)	35.84 (35.61–36.08)
*P* for difference		<.001
Elevated AST		
Yes	7920 (3.9)	61.66 (60.35–62.97)
No	193,696 (96.1)	38.54 (38.30–38.78)
*P* for difference		<.001

NOTE. Percentages may not add to 100% because of rounding.

ALT = alanine aminotransferase, AST = aspartate transaminase, CI = confidence interval, MASLD = metabolic-associated steatotic liver disease.

**Figure 1.**
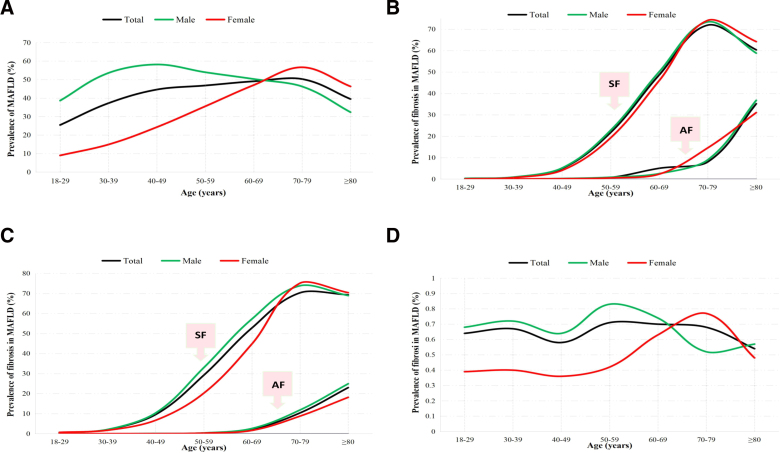
Sex-specific age trajectories in the prevalence of MASLD and hepatic fibrosis. (A) Age and sex patterns in MASLD prevalence; (B) Age and sex patterns in SF and AF prevalence by FIB-4 index; (C) Age and sex patterns in SF and AF prevalence by NFS index; (D) Age and sex patterns in AF prevalence by APRI index. AF = advanced fibrosis, APRI = AST to platelet ratio, MASLD = metabolic-associated steatotic liver disease, NFS = NAFLD fibrosis score, SF = significant fibrosis.

To elucidate the dynamic patterns of MASLD across the life course, we analyzed age and sex-specific prevalence, which revealed distinctive epidemiological trajectories (Fig. 1A). Males demonstrated an early peak in mid-life (58.15% at 40–49 years), followed by a progressive decline. In contrast, females exhibited a continuous rise in prevalence with advancing age, reaching comparable levels to males only in later life (56.63% at 70–79 years). This divergent pattern resulted in a striking crossover effect: MASLD prevalence was consistently higher in males across all age groups below 60 years, whereas females showed higher prevalence in all age groups from 70 years onward.

### 3.3. Burden and distribution of liver fibrosis in MASLD

The estimated prevalence of NIT-defined liver fibrosis in MASLD patients varied significantly by the NIT used (Table 3), demonstrating substantial disagreement between these tools. The proportion of patients with significant fibrosis (SF) was broadly similar between FIB-4 (19.12%) and NFS (21.32%). In contrast, the prevalence of AF was lowest when defined by APRI (0.61%), compared to FIB-4 (1.79%) and NFS (1.97%). Subgroup analyses identified several consistent, strong correlates. Age ≥ 60 years, elevated ALT, and elevated AST were uniformly associated with significantly higher prevalence of both SF and AF fibrosis across all 3 indices (all *P* < .001). Obesity and hypertension also showed predominantly positive associations with fibrosis prevalence, particularly when defined by NFS.

**Table 3:** Prevalence of significant fibrosis (SF) and advanced fibrosis (AF) in the MASLD population and subpopulations.

Populations	No. (%)	Prevalence, % (95% CI)				
		SF		AF		
		FIB-4	NFS	FIB-4	NFS	APRI
Overall	84,692	19.12 (18.90–19.34)	21.32 (21.07–21.56)	1.79 (1.69–1.90)	1.97 (1.86–2.07)	0.61 (0.54–0.67)
Sex						
Male	62,847 (74.2)	19.33 (19.08–19.58)	23.36 (23.09–23.63)	1.78 (1.66–1.90)	2.05 (1.92–2.17)	0.71 (0.63–0.78)
Female	21,845 (25.8)	18.90 (18.53–19.28)	19.22 (18.82–19.62)	1.80 (1.63–1.98)	1.89 (1.71–2.06)	0.50 (0.39–0.62)
*P* for difference		.163	**<.001**	.846	.149	**.001**
Age, yr						
<60	66,132 (78.1)	6.93 (6.74–7.11)	9.69 (9.47–9.91)	0.27 (0.23–0.31)	0.11 (0.08–0.14)	0.56 (0.49–0.64)
≥60	18,560 (21.9)	58.28 (57.54–59.02)	58.66 (57.92–59.41)	6.70 (6.28–7.12)	7.93 (7.48–8.37)	0.75 (0.61–0.89)
*P* for difference		**<.001**	**<.001**	**<.001**	**<.001**	**.003**
Obesity						
Yes	64,242 (75.9)	18.81 (18.54–19.07)	22.64 (22.34–22.94)	1.76 (1.64–1.88)	2.27 (2.13–2.41)	0.66 (0.57–0.74)
No	20,450 (24.1)	19.57 (19.15–19.99)	17.93 (17.51–18.34)	1.91 (1.71–2.10)	1.21 (1.04–1.38)	0.47 (0.35–0.60)
*P* for difference		.012	**<.001**	.164	**<.001**	**.002**
Diabetes						
Yes	10,048 (11.9)	18.88 (18.14–19.62)	38.74 (37.53–39.94)	1.98 (1.74–2.22)	3.74 (3.45–4.04)	2.85 (1.58–4.14)
No	74,644 (88.1)	19.34 (19.09–19.59)	18.94 (18.69–19.20)	1.76 (1.64–1.88)	1.43 (1.33–1.54)	0.47 (0.42–0.53)
*P* for difference		.274	**<.001**	.116	**<.001**	**<.001**
Hypertension						
Yes	36,239 (42.8)	19.29 (18.96–19.62)	22.64 (22.27–23.01)	1.83 (1.71–1.96)	2.07 (1.94–2.20)	0.96 (0.67–1.25)
No	48,453 (57.2)	19.10 (17.89–20.32)	20.29 (19.89–20.70)	1.80 (1.56–2.03)	1.78 (1.54–2.02)	0.53 (0.43–0.64)
*P* for difference		.488	**<.001**	.747	**.002**	**<.001**
Dyslipidemia						
Yes	62,806 (74.2)	18.55 (18.29–18.82)	21.05 (20.75–21.35)	1.58 (1.46–1.70)	1.74 (1.62–1.87)	0.65 (0.55–0.76)
No	21,886 (25.8)	20.89 (20.45–21.33)	22.32 (21.87–22.78)	2.26 (2.06–2.47)	2.42 (2.22–2.63)	0.57 (0.46–0.67)
*P* for difference		**<.001**	**<.001**	**<.001**	**<.001**	.206
Elevated ALT						
Yes	18,308 (21.6)	21.84 (20.97–22.71)	20.07 (19.24–20.89)	5.09 (4.49–5.69)	2.16 (1.68–2.64)	4.10 (3.51–4.61)
No	66,384 (78.4)	18.58 (18.35–18.81)	21.43 (21.18–21.69)	1.51 (1.41–1.61)	1.96 (1.85–2.07)	0.09 (0.05–0.12)
*P* for difference		**<.001**	**<.001**	**<.001**	**<.001**	**<.001**
Elevated AST						
Yes	5469 (6.5)	31.95 (30.60–33.29)	24.88 (23.64–26.12)	10.67 (9.76–11.59)	3.65 (3.00–4.31)	9.81 (8.74–10.89)
No	79,223 (93.5)	18.38 (18.16–18.60)	21.10 (20.85–21.34)	1.39 (1.29–1.48)	1.89 (1.78–2.00)	0.07 (0.04–0.10)
*P* for difference		**<.001**	**<.001**	**<.001**	**<.001**	**<.001**

NOTE. Percentages may not add to 100% because of rounding.

AF = advanced fibrosis, ALT = alanine aminotransferase, APRI = AST to platelet ratio, AST = aspartate transaminase, CI = confidence interval, MASLD = metabolic-associated steatotic liver disease, NFS = NAFLD Fibrosis Score, SF = significant fibrosis.

A key finding was the differential association of diabetes with fibrosis depending on the NIT: Diabetes was strongly associated with NFS-defined fibrosis (SF: 38.74%; AF: 3.74%) and APRI-AF (2.85%), but showed no significant association with FIB-4-based estimates. Conversely, dyslipidemia was associated with lower rates of FIB-4 and NFS-defined fibrosis but showed no significant association with APRI-AF. The prevalence of both SF and AF demonstrated a pronounced age-dependent rise across all noninvasive indices, with the most marked increases occurring after age 50 and peaking in the 70 to 79-year age group for SF, whereas AF prevalence by FIB-4 and NFS demonstrated a continued rise into the oldest age group. APRI showed a more modest age-related increase. These patterns are visually detailed in Figure 1B–D, which illustrate the close epidemiological link between MASLD and fibrosis risk across the lifespan, as well as the varying trajectories captured by different NITs.

### 3.4. Associated factors for MASLD and liver fibrosis

Multivariable analyses identified key associated factors for MASLD in the overall population. As shown in Table 4, in fully adjusted models, obesity demonstrated the strongest association (adjusted OR: 7.69, 95% CI: 7.52–7.87), followed by dyslipidemia (adjusted OR: 2.71, 95% CI: 2.65–2.77), diabetes (adjusted OR: 2.02, 95% CI: 1.93–2.11), and male sex (adjusted OR: 1.31, 95% CI: 1.28–1.35). These findings underscore the central role of metabolic dysfunction in MASLD pathogenesis.

**Table 4:** Multivariable logistic regression analysis for associated factors of MASLD.

Predictors	MASLD		
	Unadjusted	Model 1 OR (95% CI)	Model 2 OR (95% CI)
Male	2.97 (2.91–3.02)	2.94 (2.88–3.00)	1.31 (1.28–1.35)
Age, per 10 yr-increment	1.19 (1.18–1.20)	1.18 (1.17–1.19)	1.08 (1.07–1.08)
Obesity	10.82 (10.59–11.05)	9.35 (9.15–9.55)	7.69 (7.52–7.87)
Diabetes	3.17 (3.06–3.28)	2.32 (2.24–2.41)	2.02 (1.93–2.11)
Hypertension	2.87 (2.82–2.93)	2.32 (2.27–2.37)	1.52 (1.49–1.56)
Dyslipidemia	4.06 (3.98–4.14)	3.50 (3.43–3.57)	2.71 (2.65–2.77)
Elevated ALT	4.82 (4.68–4.97)	4.47 (4.33–4.61)	2.80 (2.69–2.92)
Elevated AST	3.22 (3.07–3.38)	2.98 (2.84–3.13)	0.91 (0.85–0.97)

Model 1: adjusted for age and sex; Model 2: adjusted for age, sex, obesity, diabetes, hypertension, dyslipidemia, elevated ALT, and elevated AST.

ALT = alanine aminotransferase, AST = aspartate transaminase, CI = confidence interval, MASLD = metabolic-associated steatotic liver disease, OR = odds ratio.

For fibrosis risk among MASLD patients (Tables S2,S3,S4,S5 and S6, Supplemental Digital Contents 2–6, which illustrate the multivariable logistic regression for SF/AF by FIB-4/NFS/APRI), elevated AST and older age emerged as the most consistent independent factors across all fibrosis stages and assessment tools. Age per 10-year increment showed strong associations with both SF (e.g., FIB-4: adjusted OR: 4.10, 95% CI: 4.01–4.20) and AF (e.g., NFS: adjusted OR: 4.62, 95% CI: 4.34–4.91). Elevated AST demonstrated particularly potent effects for AF (FIB-4: adjusted OR: 33.33, 95% CI: 25.70–43.22; APRI: 116.06, 95% CI: 76.84–175.29). Additionally, male sex was consistently associated with higher SF fibrosis risk across all scoring systems.

We observed notable heterogeneity for other metabolic factors depending on the NIT. Both dyslipidemia and obesity showed inverse associations with FIB-4 and NFS-defined fibrosis – consistent with the prevalence patterns in Table 3 **–** while diabetes was strongly associated with NFS and APRI-defined fibrosis but not with FIB-4-based estimates. Other factors, including hypertension and elevated ALT, showed less consistent associations across different models. These patterns highlight how the constituent variables of each scoring system fundamentally shape the identified risk profiles for fibrosis progression in MASLD.

## 4. Discussion

Our large-scale study provides a comprehensive assessment of MASLD in eastern China, revealing a high prevalence within this population, distinctive sex-specific age trajectories, and, critically, important discordance in current noninvasive fibrosis risk stratification that is dependent on patient subgroups and the specific NIT employed. The standardized MASLD prevalence of 39.42% reflects a severe public health challenge, consistent with rates in the US (39.1%)^[15]^ and Germany (37%).^[16]^ This aligns with the Global Burden of Disease Study, showing a ~68% increase in Chinese MASLD cases (2005–2019),^[17]^ projecting over 314 million cases by 2030.^[18]^ The high prevalence under MASLD criteria likely reflects its more inclusive, metabolism-focused definition.

A particularly striking finding was the dynamic relationship between sex, age, and MASLD risk. We observed a clear male predominance in overall MASLD prevalence (49.98% vs 28.59%), consistent with global epidemiology.^[19]^ However, our age-stratified analysis reveals a more nuanced pattern: while males demonstrate an early peak in mid-life followed by gradual decline, females exhibit a steady increase with age, surpassing male prevalence after age 70. This sexually dimorphic trajectory is consistent with a protective role of estrogen in MASLD pathogenesis. The sharp rise in female MASLD prevalence after age 50 coincides with the menopausal transition, suggesting that declining estrogen levels may be a key driver.^[20–22]^

The relationship between adiposity and MASLD risk demonstrated a strong association in our population, with obesity emerging as the strongest independent factor (adjusted OR: 7.69). This is consistent with numerous epidemiological studies linking rising obesity rates with increasing MASLD prevalence.^[19]^ The profound impact of severe obesity on MASLD risk underscores the critical importance of weight management strategies. Current MASLD management guidelines emphasize lifestyle interventions as foundational therapy.^[23]^ Our findings reinforce that weight management represents a crucial intervention point for addressing the growing MASLD epidemic.

Accurate fibrosis assessment is crucial for risk stratification in MASLD. Our findings reveal substantial discordance across NITs, which presents a clinical challenge for interpreting these test results in practice. Although guidelines recommend FIB-4,^[1,6]^ our data show significant limitations in MASLD, especially in patients with metabolic comorbidities. This divergence highlights a key methodological issue: FIB-4, NFS, and APRI were developed predominantly in Western NAFLD clinic cohorts.^[7,8]^ Their external validity, when applied to an Asian, MASLD-defined, community-based health checkup population, may be limited. Our findings, showing marked disagreement between tools, especially in patients with diabetes, suggest that the risk estimates and clinical interpretation of these tools may require cautious application and potential re-calibration for the contemporary MASLD population.

The most striking observation was the marked heterogeneity in NIT performance among patients with diabetes. While NFS and APRI consistently identified diabetes as a significant factor for fibrosis, FIB-4 failed to do so. This critical discrepancy points to fundamental limitations of applying a liver-disease-agnostic tool to the unique pathophysiology of diabetic MASLD. We propose that the metabolic milieu of type 2 diabetes creates a physiological context that systematically undermines the assumptions of the FIB-4 index. First, the well-documented pro-inflammatory and pro-thrombotic state in diabetes can stimulate thrombopoiesis, leading to elevated platelet counts,^[24]^ which artificially lowers the FIB-4 score. Second, in advanced MASLD with diabetic metabolic dysregulation, a disconnect may develop between transaminase levels and the actual fibrotic burden, rendering FIB-4 less sensitive in this high-risk population.^[19]^ Therefore, the performance gap between NITs reflects the complex interplay between systemic metabolic dysfunction and hepatic fibrogenesis, which tools like NFS – by incorporating metabolic parameters – are inherently better equipped to capture.

Our findings reveal a distinct methodological gradient in the estimated prevalence of NIT-defined AF in MASLD. Our NIT results for AF (FIB-4: 1.79%; NFS: 1.97%; APRI: 0.61%) align with a meta-analysis from general population studies.^[25]^ It is important to note that these estimates are based on noninvasive surrogates and may not reflect the true prevalence of histologically confirmed fibrosis, which is typically higher in biopsy cohorts (>30%).^[26]^ This spectrum mirrors the ascending severity of the MASLD spectrum across populations. Notably, the prevalence of AF defined by APRI was the lowest (0.61%), likely reflecting its lower sensitivity for detecting AF in MASLD, particularly in a community cohort with mildly elevated transaminases, as it was originally developed for hepatitis C.^[27]^ This highlights that different NITs capture different aspects of fibrosis risk and are not interchangeable.

Beyond test-specific considerations, the consistent identification of advancing age as a powerful factor across all NITs underscores the cumulative nature of liver injury in MASLD.^[28]^ The mechanisms underlying this age-dependent risk are multifactorial.^[29]^ Similarly, the strong association between AST elevation and AF reflects not only hepatocellular injury but also its emerging role as a marker of mitochondrial dysfunction, which can drive fibrogenesis.^[30]^ Although age is a component of FIB-4 and NFS, its strong association with APRI-defined fibrosis (which does not include age) supports that advancing age is a genuine biological risk factor.

Our findings reveal complex relationships between metabolic factors and fibrosis risk. While obesity demonstrated the strongest association with MASLD development, its relationship with fibrosis progression varied significantly depending on the assessment tool used. This discrepancy may reflect distinct metabolic phenotypes among obese patients,^[31]^ where adipose tissue distribution patterns may be more relevant to fibrogenesis than BMI alone.^[32]^ Hypertension consistently showed positive associations with fibrosis, particularly with NFS, aligning with the recognized role of the liver-angiotensin system.^[33]^ The paradoxical inverse associations between dyslipidemia and fibrosis metrics may be partially explained by the widespread use of statins, which may attenuate hepatic inflammation and fibrosis progression,^[34]^ and/or by altered systemic lipid metabolism in advanced liver disease.^[35]^

Based on our findings, which highlight the discordance between NITs, we suggest that a context-aware approach to fibrosis risk assessment warrants further investigation. While current guidelines recommend FIB-4 as an initial screening tool,^[1,6]^ our data suggest that relying on a single NIT may be insufficient, particularly in patients with metabolic comorbidities such as diabetes. Future prospective studies should explore whether a sequential strategy – using FIB-4 as an initial screen, followed by NFS or vibration-controlled transient elastography for those with indeterminate results or high-risk features – improves risk stratification accuracy and clinical outcomes. Consequently, clinicians should be aware that FIB-4 should not be used in isolation to exclude AF risk, especially in MASLD patients with diabetes.

This study has limitations that also illuminate paths for future research. First, our MASLD diagnosis relied on ultrasonography, which has reduced sensitivity for mild steatosis,^[36]^ and may lead to an underestimation of the true MASLD prevalence. Second, the cross-sectional nature precludes causal inference regarding fibrosis progression. Future research should focus on developing dynamic, MASLD-specific risk prediction models. Third, our single-center, health-checkup-based sample may affect generalizability to the general population of Eastern China, as health checkup attendees may be healthier and of higher socioeconomic status than the general population; multi-center, population-based studies are warranted. Fourth, the absence of a gold standard (liver biopsy or MRI elastography) means that our findings describe NIT-defined risk rather than histologically confirmed fibrosis. Future studies incorporating these modalities are essential to validate the performance of these NITs in Chinese MASLD populations. Finally, the lack of comprehensive medication history limits our ability to fully adjust for potential confounders, such as the impact of statins on lipid levels and potentially on fibrosis scores.

## 5. Conclusion

In conclusion, our findings describe the substantial burden of MASLD and NIT-defined fibrotic risk in a large health checkup cohort from eastern China and critically assess the agreement between current noninvasive assessment tools. We demonstrate that these tools provide discordant, rather than congruent, risk estimates, reflecting the influence of their constituent variables and the metabolic heterogeneity of MASLD. These insights highlight the need for cautious interpretation of single NIT results and underscore the importance of a more sophisticated, context-aware approach to fibrosis risk stratification that considers the choice of NIT alongside the patient’s metabolic profile, suggesting directions for more personalized and effective management strategies. Future prospective cohort studies are warranted to validate these findings and to develop dynamic, MASLD-specific risk prediction models incorporating longitudinal data and hard clinical outcomes.

## Acknowledgments

We thank Dr Yuan Gao, PhD in Epidemiology, from the Affiliated Hospital of Xuzhou Medical University, for providing statistical consultation and expertise in the analysis and interpretation of the data. Dr Yuan Gao has provided written consent to be named in the acknowledgements.

## Author contributions

**Conceptualization:** Mingxing Chang, Peipu Shen, Guifang Shen.

**Data curation:** Mingxing Chang.

**Formal analysis:** Mingxing Chang, Peipu Shen.

**Funding acquisition:** Mingxing Chang.

**Investigation:** Mingxing Chang.

**Methodology:** Mingxing Chang, Peipu Shen, Guifang Shen.

**Software:** Mingxing Chang.

**Validation:** Peipu Shen, Guifang Shen.

**Writing – original draft:** Mingxing Chang.

**Writing – review & editing:** Guifang Shen.

**Figure:**