# Supplementary material for: Epidemiology of metabolic dysfunction-associated steatotic liver disease and discordance in non-invasive fibrosis scores in Eastern China: A cross-sectional study
Source: Medicine (Baltimore). 2026 Jun 5;105(23):e49110. doi: 10.1097/MD.0000000000049110 (PMC13246051; doi:10.1097/MD.0000000000049110)
Supplement: Supplementary file 1 [file medi-105-e49110-s002.docx]

**Supplemental Digital Content 2**

Table S2 Multivariable logistic regression analysis for associated factors of significant fibrosis (SF) in patients with MASLD, evaluated by the FIB-4 index

| Predictors | MASLD | | |
| --- | --- | --- | --- |
|  | Unadjusted | Model 1 OR (95% CI) | Model 2 OR (95% CI) |
| Male | 0.67 (0.64-0.69) | 1.24 (1.19-1.30) | 1.27 (1.21-1.33) |
| Age, per 10 y-increment | 3.80 (3.72-3.88) | 3.86 (3.78-3.95) | 4.10 (4.01-4.20) |
| Obesity | 0.79 (0.76-0.82) | 0.95 (0.91-1.00) | 0.92 (0.88-0.97) |
| Diabetes | 2.12 (2.02-2.22) | 0.89 (0.84-0.94) | 0.84 (0.80-0.89) |
| Hypertension | 2.41 (2.33-2.50) | -- | -- |
| Dyslipidemia | 0.75 (0.72-0.78) | 0.81 (0.77-0.85) | 0.78 (0.75-0.82) |
| Elevated ALT | 0.52 (0.50-0.55) | 1.60 (1.51-1.70) | 0.84 (0.78-0.91) |
| Elevated AST | 1.71 (1.61-1.82) | 4.99 (4.60-5.41) | 5.97 (5.39-6.61) |

NOTE. --indicates that the variable was not included in the model.

Model 1: adjusted for age and sex; Model 2: adjusted for age, sex, obesity, diabetes, hypertension, dyslipidemia, elevated ALT and elevated AST.

Abbreviations: ALT, alanine aminotransferase; AST, aspartate transaminase; CI, confidence interval; MASLD, metabolic-associated steatoticy liver disease; OR, odds ratio; SF, significant fibrosis.
