# Supplementary material for: Epidemiology of metabolic dysfunction-associated steatotic liver disease and discordance in non-invasive fibrosis scores in Eastern China: A cross-sectional study
Source: Medicine (Baltimore). 2026 Jun 5;105(23):e49110. doi: 10.1097/MD.0000000000049110 (PMC13246051; doi:10.1097/MD.0000000000049110)
Supplement: Supplementary file 2 [file medi-105-e49110-s003.docx]

**Supplemental Digital Content 3**

Table S3 Multivariable logistic regression analysis for associated factors of advanced fibrosis (AF) in patients with MASLD, evaluated by the FIB-4 index.

| Predictors | MASLD | | |
| --- | --- | --- | --- |
|  | Unadjusted | Model 1 OR (95% CI) | Model 2 OR (95% CI) |
| Male | 0.70 (0.62-0.79) | 1.21 (1.07-1.37) | 1.28 (1.12-1.46) |
| Age, per 10 y-increment | 3.40 (3.24-3.57) | 3.43 (3.26-3.60) | 3.88 (3.67-4.10) |
| Obesity | 0.76 (0.67-0.86) | -- | 0.80 (0.70-0.92) |
| Diabetes | 2.67 (2.34-3.03) | 1.18 (1.03-1.35) | -- |
| Hypertension | 3.17 (2.81-3.59) | -- | -- |
| Dyslipidemia | 0.53 (0.47-0.60) | 0.69 (0.61-0.78) | 0.61 (0.53-0.69) |
| Elevated ALT | 1.32 (1.16-1.50) | 5.25 (4.53-6.09) | 0.58 (0.45-0.76) |
| Elevated AST | 7.17 (6.33-8.11) | 20.50 (17.57-23.93) | 33.33 (25.70-43.22) |

NOTE. --indicates that the variable was not included in the model.

Model 1: adjusted for age and sex; Model 2: adjusted for age, sex, obesity, diabetes, hypertension, dyslipidemia, elevated ALT and elevated AST.

Abbreviations: AF, advanced fibrosis; ALT, alanine aminotransferase; AST, aspartate transaminase; CI, confidence interval; MASLD, metabolic-associated steatotic liver disease; OR, odds ratio.
