# Supplementary material for: Epidemiology of metabolic dysfunction-associated steatotic liver disease and discordance in non-invasive fibrosis scores in Eastern China: A cross-sectional study
Source: Medicine (Baltimore). 2026 Jun 5;105(23):e49110. doi: 10.1097/MD.0000000000049110 (PMC13246051; doi:10.1097/MD.0000000000049110)
Supplement: Supplementary file 3 [file medi-105-e49110-s004.docx]

**Supplemental Digital Content 4**

Table S4 Multivariable logistic regression analysis for associated factors of significant fibrosis (SF) in patients with MASLD, evaluated by the NFS

| Predictors | MASLD | | |
| --- | --- | --- | --- |
|  | Unadjusted | Model 1 OR (95% CI) | Model 2 OR (95% CI) |
| Male | 0.89 (0.86-0.93) | 1.75 (1.68-1.83) | 1.51 (1.45-1.59) |
| Age, per 10 y-increment | 3.11 (3.05-3.17) | 3.26 (3.20-3.32) | 3.07 (3.01-3.13) |
| Obesity | 1.23 (1.18-1.27) | 1.67 (1.59-1.74) | 1.79 (1.71-1.88) |
| Diabetes | 6.89 (6.59-7.20) | 4.79 (4.55-5.04) | 4.98 (4.72-5.24) |
| Hypertension | 2.66 (2.58-2.75) | 1.26 (1.21-1.31) | 1.14 (1.09-1.19) |
| Dyslipidemia | 0.85 (0.82-0.89) | 0.93 (0.89-0.97) | 0.85 (0.81-0.89) |
| Elevated ALT | 0.41 (0.39-0.43) | 0.92 (0.87-0.97) | 0.61 (0.57-0.66) |
| Elevated AST | 0.93 (0.87-0.99) | 1.64 (1.51-1.77) | 1.99 (1.80-2.20) |

NOTE. --indicates that the variable was not included in the model.

Model 1: adjusted for age and sex; Model 2: adjusted for age, sex, obesity, diabetes, hypertension, dyslipidemia, elevated ALT and elevated AST.

Abbreviations: ALT, alanine aminotransferase; AST, aspartate transaminase; CI, confidence interval; MASLD, metabolic-associated steatotic liver disease; NFS, NAFLD Fibrosis Score; OR, odds ratio; SF, significant fibrosis.
