# Supplementary material for: Epidemiology of metabolic dysfunction-associated steatotic liver disease and discordance in non-invasive fibrosis scores in Eastern China: A cross-sectional study
Source: Medicine (Baltimore). 2026 Jun 5;105(23):e49110. doi: 10.1097/MD.0000000000049110 (PMC13246051; doi:10.1097/MD.0000000000049110)
Supplement: Supplementary file 4 [file medi-105-e49110-s005.docx]

**Supplemental Digital Content 5**

Table S5 Multivariable logistic regression analysis for associated factors of advanced fibrosis (AF) in patients with MASLD, evaluated by the NFS

| Predictors | MASLD | | |
| --- | --- | --- | --- |
|  | Unadjusted | Model 1 OR (95% CI) | Model 2 OR (95% CI) |
| Male | 0.74 (0.65-0.83) | 1.37 (1.21-1.56) | 1.18 (1.04-1.35) |
| Age, per 10 y-increment | 4.76 (4.50-5.04) | 4.81 (4.54-5.09) | 4.62 (4.34-4.91) |
| Obesity | 1.73 (1.49-2.01) | 2.35 (1.99-2.77) | 2.66 (2.25-3.15) |
| Diabetes | 6.80 (6.08-7.61) | 3.32 (2.93-3.76) | 3.68 (3.24-4.18) |
| Hypertension | 4.40 (3.86-5.01) | 1.26 (1.09-1.45) | -- |
| Dyslipidemia | 0.50 (0.45-0.56) | 0.68 (0.60-0.77) | 0.63 (0.56-0.72) |
| Elevated ALT | 0.28 (0.23-0.35) | -- | 0.35 (0.25-0.50) |
| Elevated AST | 1.33 (1.09-1.63) | 2.58 (2.06-3.25) | 5.08 (3.64-7.09) |

NOTE. --indicates that the variable was not included in the model.

Model 1: adjusted for age and sex; Model 2: adjusted for age, sex, obesity, diabetes, hypertension, dyslipidemia, elevated ALT and elevated AST.

Abbreviations: AF, advanced fibrosis; ALT, alanine aminotransferase; AST, aspartate transaminase; CI, confidence interval; MASLD, metabolic-associated steatotic liver disease; NFS, NAFLD Fibrosis Score; OR, odds ratio.
