# Supplementary material for: Epidemiology of metabolic dysfunction-associated steatotic liver disease and discordance in non-invasive fibrosis scores in Eastern China: A cross-sectional study
Source: Medicine (Baltimore). 2026 Jun 5;105(23):e49110. doi: 10.1097/MD.0000000000049110 (PMC13246051; doi:10.1097/MD.0000000000049110)
Supplement: Supplementary file 5 [file medi-105-e49110-s006.docx]

**Supplemental Digital Content 6**

Table S6 Multivariable logistic regression analysis for associated factors of advanced fibrosis (AF) in patients with MASLD, evaluated by the APRI.

| Predictors | MASLD | | |
| --- | --- | --- | --- |
|  | Unadjusted | Model 1 OR (95% CI) | Model 2 OR (95% CI) |
| Male | 1.39 (1.13-1.71) | 1.39 (1.13-1.71) | -- |
| Age, per 10 y-increment | -- | -- | 1.29 (1.21-1.38) |
| Obesity | 1.45 (1.17-1.79) | 1.38 (1.11-1.72) | -- |
| Diabetes | 3.36 (2.80-4.02) | 3.55 (2.93-4.30) | 2.17 (1.78-2.65) |
| Hypertension | 1.75 (1.48-2.07) | 1.73 (1.46-2.04) | -- |
| Dyslipidemia | -- | -- | 0.62 (0.50-0.76) |
| Elevated ALT | 37.50 (28.11-50.02) | 57.62 (42.83-77.51) | 2.64 (1.79-3.89) |
| Elevated AST | 204.22 (148.42-281.00) | 237.48 (172.17-327.56) | 116.06 (76.84-175.29) |

NOTE. --indicates that the variable was not included in the model.

Model 1: adjusted for age and sex; Model 2: adjusted for age, sex, obesity, diabetes, hypertension, dyslipidemia, elevated ALT and elevated AST.

Abbreviations: AF, advanced fibrosis; ALT, alanine aminotransferase; APRI, AST to platelet ratio; AST, aspartate transaminase; CI, confidence interval; MASLD, metabolic-associated steatotic liver disease; OR, odds ratio.
